# Clinical Trial Electronic Portals for Expedited Safety Reporting: Recommendations from the Clinical Trials Transformation Initiative Investigational New Drug Safety Advancement Project

**DOI:** 10.2196/cancer.6701

**Published:** 2016-12-15

**Authors:** Raymond P Perez, Shanda Finnigan, Krupa Patel, Shanell Whitney, Annemarie Forrest

**Affiliations:** ^1^ The University of Kansas Cancer Center Kansas City, KS United States; ^2^ National Cancer Institute National Institutes of Health Bethesda, MD United States; ^3^ Merck Research Laboratories Sudbury, MA United States; ^4^ Eli Lilly and Company Indianapolis, IN United States; ^5^ Clinical Trials Transformation Initiative Durham, NC United States

**Keywords:** clinical trials, investigational new drug application, risk management

## Abstract

**Background:**

Use of electronic clinical trial portals has increased in recent years to assist with sponsor-investigator communication, safety reporting, and clinical trial management. Electronic portals can help reduce time and costs associated with processing paperwork and add security measures; however, there is a lack of information on clinical trial investigative staff’s perceived challenges and benefits of using portals.

**Objective:**

The Clinical Trials Transformation Initiative (CTTI) sought to (1) identify challenges to investigator receipt and management of investigational new drug (IND) safety reports at oncologic investigative sites and coordinating centers and (2) facilitate adoption of best practices for communicating and managing IND safety reports using electronic portals.

**Methods:**

CTTI, a public-private partnership to improve the conduct of clinical trials, distributed surveys and conducted interviews in an opinion-gathering effort to record investigator and research staff views on electronic portals in the context of the new safety reporting requirements described in the US Food and Drug Administration’s final rule (Code of Federal Regulations Title 21 Section 312). The project focused on receipt, management, and review of safety reports as opposed to the reporting of adverse events.

**Results:**

The top challenge investigators and staff identified in using individual sponsor portals was remembering several complex individual passwords to access each site. Also, certain tasks are time-consuming (eg, downloading reports) due to slow sites or difficulties associated with particular operating systems or software. To improve user experiences, respondents suggested that portals function independently of browsers and operating systems, have intuitive interfaces with easy navigation, and incorporate additional features that would allow users to filter, search, and batch safety reports.

**Conclusions:**

Results indicate that an ideal system for sharing expedited IND safety information is through a central portal used by all sponsors. Until this is feasible, electronic reporting portals should at least have consistent functionality. CTTI has issued recommendations to improve the quality and use of electronic portals.

## Introduction

Safety reporting is a necessary element of clinical trials to help ensure patient safety during the investigation of a new drug or medical device. With advances in technology, safety reporting, along with other clinical trial data reporting, is moving to electronic formats from being largely paper-based. To encourage electronic submissions and integration of other technological capabilities into trial document management, the US Food and Drug Administration (FDA) has previously released guidance on electronic submissions and source data [1,2].

Aligned with this progress is the increased use of electronic portals to facilitate communication between sponsors and the investigative staff during clinical trials. Few publications in the scholarly literature have focused on portals; however, it is acknowledged within the clinical research field that use of portals is proliferating. Typically, clinical trial portals are developed by sponsors or contract research organizations (CRO) to provide a centralized location for trial-specific documents and information (eg, regulatory and safety documents, protocols, investigator brochures). Using clinical trial portals can reduce the time and cost associated with processing paperwork, among other advantages. The portal also provides increased security with document management and communication through the use of log-in identification and password protection. Once the portals are developed, investigators are given access to the portal through specific log-in credentials. Investigators can access trial-specific documents provided by the sponsor or CRO. Throughout the trial, investigators periodically log in to review safety reports.

The Clinical Trials Transformation Initiative (CTTI) is a public-private partnership whose mission is to develop and drive adoption of practices that will increase the quality and efficiency of clinical trials. CTTI initiated the investigation new drug (IND) Safety Advancement Project to investigate barriers to implementation of the FDA final rule on expedited IND safety reporting (Code of Federal Regulations [CFR] Title 21 Part 312) and propose solutions to address identified barriers, from the perspective of both investigators and sponsors. The project focused specifically on oncology research, where these issues tend to be most acute.

Evidence gathered during the project indicated that investigators have difficulty using clinical trial portals and that portals may contribute to confusion and burden investigators’ experience related to the IND safety reporting system. Therefore, the CTTI project team sought to identify best practices for managing IND safety reports using electronic portals and formulated recommendations based on data collected through evidence-gathering activities.

## Methods

### Approach

The IND Safety Advancement Project team included 20 individuals representing a wide range of stakeholders, including industry, academic institutions, institutional review board (IRB), regulatory, patient advocate, and other perspectives; all groups were considered equal partners. The primary focus of the project was to investigate barriers related to the lack of implementation of the final rule (21 CFR 312) and provide recommendations for better compliance; however, a portion of this project was specifically devoted to addressing the use of clinical trial portals. The team employed 3 main research strategies to gather evidence: surveys, expert interviews, and an expert meeting.

### Survey

An online survey was designed to assess challenges related to cancer researcher management of IND safety reporting processes, with a subset of questions about the specific challenges related to use of electronic portals to manage safety reports (Textbox 1). The survey was distributed to contact networks via CTTI, the American Society of Clinical Oncology, the American Association of Cancer Institutes, and the US Oncology Network. Recipients were encouraged to freely forward the survey to colleagues; because no data is available describing the number of potential respondents who had access to the survey, a formal response rate cannot be determined. To establish face validity, the survey was reviewed and pilot-tested on a subset of the intended population, but no formal validation or internal consistency checks were performed. Participation was voluntary, anonymous, and uncompensated. The survey was distributed via Constant Contact, an online marketing company, and completed through Qualtrics. Survey data collected from November 18, 2014, through December 30, 2014, were aggregated by the Duke Center for Learning Health Care and distributed to project team members for descriptive analysis. The complete survey can be viewed online [3].

**Survey questions on clinical trial portals.**

Questions on current issues:

- If IND safety reports are distributed via a sponsor safety reporting portal, do you have difficulty accessing the IND safety reporting portal?
- Please describe the difficulty you have accessing the IND safety reporting portal.

Questions on suggestions for improvement:

- What things about the current IND safety reporting system should be changed?
- If you were starting from scratch, what would an ideal IND safety reporting system look like?

### Qualitative Interviews

A total of 20 in-depth, hour-long interviews were conducted by a professional interviewer as an opinion-gathering effort to more fully understand sponsor and investigative staff perspectives on the management of IND safety reporting processes. Interview participants who were considered experts in the topic were approached by CTTI. Survey respondents were also able to volunteer for interview participation. In January and February 2015, 13 individuals representing investigative staff working on oncology clinical trials and 7 pharmacovigilance leaders from 5 large global pharmaceutical companies were interviewed. Prepared questions were included in an interview guide [4]. The goal was to understand the receipt and management of safety reports. None of the questions explicitly asked about the desired features of clinical trial portals; however, interview respondents were free to comment on their experiences with trial portals and were encouraged to elaborate on current challenges and suggestions for improvement.

The project, including the surveys and interview guides, was designated as exempt research by the Duke University IRB.

### Expert Meeting

The IND Safety Advancement Project team analyzed survey results and interviews, developed draft recommendations based on responses, and presented this information at a 2-day expert meeting in July 2015 attended by 47 individuals representing a variety of clinical trial stakeholders. Discussion from the meeting was used by the project team to refine the recommendations through iterative, consensus-driven discussion, and they were approved by CTTI’s Executive Committee prior to official release (December 2015) [5]. Approximately half of 1 meeting day was devoted to discussing the common problems with and desired features for clinical trial portals. A summary of the expert meeting is available online [6].

## Results

### Overview

The survey had 201 respondents. The majority of the respondent population had academic or community-based research backgrounds with more than 10 years of clinical trial experience. A majority of the respondent-affiliated organizations conducted more than 30 studies concurrently and represented all phases of clinical trial development sponsored by industry and government. Full results of the survey and interviews are being reported concurrently elsewhere [7]. This manuscript focuses only on data related to clinical trial portal use.

### Current Issues

Responses from investigators and other study staff indicate that 80% (33/41) of investigators and 92% (133/144) of study staff received IND safety reports through portals. Approximately half (21/41, 51%) of investigators and 44% (64/144) of staff reported difficulty accessing sponsors’ IND safety reporting portals. When survey respondents were asked to provide free-text responses specifying difficulties encountered accessing a portal, a common answer was the problem of remembering passwords for numerous individual sponsor portals. Other difficulties voiced by respondents were challenges with operating systems, software compatibility, and differing application versions. Respondents indicated that many interfaces are difficult to navigate and do not incorporate intuitive design elements. Additionally, many portals have slow processing times, and their applications often crash or fail. For these reasons, downloading reports can be time-consuming for the investigative staff. Finally, respondents noted that generic email notifications are not particularly helpful and many choose to block these emails. The top-rated challenges were as follows:

- Remembering numerous, complex passwords for individual sponsor portals
- Encountering problems related to different operating systems, software compatibility, and application versioning
- Navigating through nonintuitive user interfaces and slow sites
- Encountering log-in or site issues due to investigative staff turnover
- Receiving too many generic email notifications
- Time-consuming process of downloading reports

Although distribution of streamlined reports via electronic portals was intended to improve the efficiency of the safety reporting process, interviewees from investigative sites reported that they continue to receive an unmanageable volume of IND safety reports. Active sites described the volume of reports as “staggering.”

### Beneficial and Desired Features

Respondents described a number of benefits related to use of portals for safety reporting, including automatic notifications of trends or unexpected adverse events, which help guide treatment decisions for patients. Investigative staff indicated that it was easier to identify risks when reports were submitted through the portal. Summary reporting and the defined attribution and causality available on the portal can help filter the safety information for study staff. With enhanced signal detection, the investigative staff can identify information that may generate important changes to the protocol or consent and help them make determinations on the utility of the study and other risk/benefit assessments. Additionally, electronic reports are more efficient and easier to retain and track.

The attributes displayed in Textbox 2 were identified as important and desired features of electronic portals based on the project team’s analysis of the survey responses, which highlighted the inconsistent functionality with current portals. These desired attributes can be categorized as improving (1) the overall system functionality, (2) the user interface, (3) report management and analysis, and (4) report notification, acknowledgement, and verification.

**Clinical Trials Transformation Initiative official recommendations for attributes of electronic portals for expedited safety reporting: categories and desired features.**

Overall system functionality:

- Cross-browser compatibility; portal works seamlessly with all commonly used browsers
- Operating system independent
- Mobile-friendly
- Quick report download time (ie, externally hosted, cloud-based)
- Simplified security management system (eg, end-user control over password management, biometric identification in lieu of passwords, and/or ability to integrate with various identity access applications)

User interface:

- Intuitive, easy-to-navigate interface requiring few clicks to access safety reports directly or via hyperlink contained in an email notification
- Flexibility within the portal for use with varied institutional processes

Report management and analysis:

- Print reports or download multiple reports with one click to a compact disc, computer, or electronic investigator site file
- Filter reports by event so follow-up safety reports do not appear as new events
- Search and display safety reports using custom dates, by country of origin and/or event name
- Export single reports as well as aggregated data
- Drill down to access single reports and write-ups
- Reports remain visible for the life of the trial

Report notification, acknowledgment, and verification:

- Ability to batch safety report notifications (per day/week) per investigative site user’s preference
- Ability for principal investigator to delegate the task of accessing safety reports via portal to another person at the site
- Easy acknowledgement of safety reports by investigative site staff (eg, click on a link to the report, check a box, or check-all option)
- Ability to send and record acknowledgement of a safety report only once across multiple trials for the same investigational product yet still show the report under each trial
- End-to-end audit trail that can be printed and saved or stored for future reference by both the sponsor and investigative site
- Ability of the sponsor to document delivery of reports within the portal if an alternative means of reporting is required (ie, the sponsor cannot access the portal and requires hard copy)
- Two-way communication between the investigative site and sponsor regarding safety reports

## Discussion

### Summary

As described elsewhere [8], investigative sites are still being inundated with individual safety reports despite new reporting requirements issued in the final rule. A priority for investigators is to identify and review important safety signals to help ensure patient safety during a trial; this can be particularly challenging when there is a large volume of lengthy paper-based reports. Electronic portals have features that can assist with filtering reports, easing the burden on investigators; however, in the opinion of the investigative staff, certain features still need improvement.

Survey results indicated that an ideal system for sharing expedited IND safety information with investigative sites is through a central portal used by all sponsors in order to improve efficiency and reduce paperwork burden (recognizing that electronic systems may not be feasible for all study sponsors). This approach may reduce the number of passwords and avoid technological issues (eg, software incompatibility) by standardizing the use of a central portal. While literature describing clinical trial portal use is limited, some reports validate survey respondents’ perceived obstacles, indicating that investigative staff and investigators struggle to recall 7 to 15 passwords per individual user [9]. Until use of a single, central portal is feasible, electronic reporting portals should at least have consistent functionality.

The attributes listed in Textbox 2 are suggested to increase portal efficiency and ease of use. As noted, important features include the following:

- Browser and operating system independence so that all users can access the portal regardless of software preferences
- An intuitive interface that is clearly labeled and provides easy navigation
- Ability to filter and search reports to quickly access only the relevant documents needed at the time
- Increased functionality to batch reports so that all files can be downloaded simultaneously
- Ability to acknowledge receipt quickly with a check-box option and to update this acknowledgement across multiple trials for the same product

Portal features to more accurately track audit trails can be particularly helpful, as reports can be categorized or searched by date, number, compound, trial number, upload/availability date, download/access date, identity of users who accessed the report, and any actions (eg, downloading, printing, saving) conducted by a specific user. As indicated in Textbox 2, notifications can be useful for study staff; however, given the high volume of reports received, some may be treated as spam. Another desired feature could provide a reminder to designate the source of the email so that it is routed and recognized appropriately. Currently, CTTI does not recommend an electronic signature requirement by the principal investigator or other investigative staff to access portal content, which is consistent with the FDA guidance.

To support appropriate use of the portal, CTTI also recommends that there be improved education for investigative sites including guidance not only on portal functionality but also regarding best practices for incorporating portal management into site report management processes. Finally, we recommend usability testing for portal-related educational material.

### Limitations

The main limitation of the study that should be acknowledged is the small sample size that was selected by convenience, which may be susceptible to bias. Additionally, survey response rate was not able to be determined. CTTI acknowledges these shortcomings and that this research is exploratory and qualitative in nature and not statistically measureable. However, change often begins with small steps, and it is our hope that this research sparks a broader discussion across the industry. CTTI encourages additional research on this topic. The perspectives described in this manuscript are descriptive; in order to be conclusive, an appropriately powered study would need to be conducted. CTTI and other independent organizations cannot require that sponsors adopt recommendations nor mandate inclusion or standardization of portal features, which is why we urge portal developers to consider the perspectives of the portal users to drive change.

Industry technology companies are investing in integrating sponsor trial portals with enhanced capabilities including interactive voice response, electronic data capture, clinical trial management systems, and investigator databases [9,10]. The ability to quickly identify and access individual reports can reduce workload burden and time spent searching considerably; however, it is important to assess how a newly introduced portal affects overall workflow of the study team. CTTI suggests that investigative site users receive more and improved education, including guidance on portal functionality in addition to best practices for incorporating portal management into site report management processes. Depending on the changes, portal management may need to be reevaluated. Finally, CTTI suggests performing usability testing for portal-related educational material in order to maximize the benefits of electronic portal use for IND safety reporting.

### Conclusions

The suggestions provided in this manuscript have been released as CTTI recommendations [5]. CTTI believes these recommendations are timely, as a number of groups are currently working on the functionality of clinical trial portals. TransCelerate BioPharma Inc is one example; as noted in their press release, they have launched “a technology that will allow clinical trial sites to streamline investigative site information and establish a central access point for interaction between the site and multiple clinical trial sponsors” [10]. It is our hope that these and similar efforts will improve clinical trial portals. These recommendations combined with this description of desired features, along with other CTTI-developed educational materials, will be disseminated to stakeholders and the public through publications, presentations, and the CTTI website. The recommendations are intended to improve clinical trial portal development, access, and functionality and to enhance user experience overall. Clinical trial portals designed to address the current barriers can also save money for sponsors because they would no longer need alternate processes for safety reporting receipt and management; could increase site user satisfaction, compliance, and tracking; and may help investigators take immediate action on patient safety issues.

## Abbreviations

CFR: Code of Federal Regulations
CRO: contract research organization
CTTI: Clinical Trials Transformation Initiative
FDA: Food and Drug Administration
IND: investigational new drug
IRB: institutional review board
